# The adverse effects of clozapine on blood markers and electrocardiographic findings in patients with schizophrenia

**DOI:** 10.37796/2211-8039.1707

**Published:** 2026-06-01

**Authors:** Papageorgiou Anastasios, Vrettou Christina, Koukouli Andriana, Kalogirou Sofia, Gkrampovari Maria, Tampaki Despoina, Bartzi Dimitra, Tsatsou Ioanna, Tasoulis Athanasios

**Affiliations:** a Internal Medicine Department, Psychiatric Hospital of Attica “Dafni”, Athens, Greece; b Second Psychiatric Department, Psychiatric Hospital of Attica “Dafni”, Athens, Greece; c Oncology Department, Hellenic Airforce General Hospital, Athens, & Medical School, University of Crete, Herakleion, Greece; d One Day Clinic, Hellenic Airforce General Hospital, Athens, Greece

**Keywords:** Clozapine, Schizophrenia, Blood markers, Electrocardiogram, Adverse effects

## Abstract

**Introduction:**

Clozapine remains the gold-standard antipsychotic for treatment-resistant schizophrenia, but its use is hindered by rare but serious adverse effects, such as hematologic abnormalities and myocarditis.

**Purpose:**

This study investigated the adverse impacts of clozapine on blood markers and electrocardiographic (ECG) findings among patients with schizophrenia, emphasizing in early detection of life-threatening complications.

**Methods:**

All patients initiated on clozapine at a psychiatric hospital in Athens, Greece, between January 2022 and June 2024 were considered for inclusion. Thirty-one patients with complete clinical and laboratory data were monitored for hematological, biochemical, and ECG changes at baseline, days 5–10, and after 1–2 months. Parameters evaluated included white blood cell (WBC) counts, differential counts, C-reactive protein (CRP), hepatic enzymes, creatine phosphokinase (CPK), troponin, and ECG changes.

**Results:**

Of the 31 patients, two (6.5%) developed clozapine-induced myocarditis within the first three weeks, confirmed by clinical symptoms, ECG abnormalities, and elevated troponin levels, necessitating immediate clozapine discontinuation. Four patients exhibited prolonged QTc, two overlapping with myocarditis cases. Eleven experienced elevated CRP, while eosinophilia and transient hepatic or muscular enzyme elevations were also common, especially during rapid titration phases; indicating inflammatory responses or mild organ involvement. No cases of agranulocytosis or severe eosinophilic complications occurred.

**Conclusions:**

Clozapine can induce significant hematologic, inflammatory, hepatic, muscular, and cardiac adverse effects, particularly during rapid titration. Systematic monitoring of blood markers and ECGs was vital in early detection and management of these complications, especially myocarditis. The findings underscore the importance of cautious titration protocols and tailored surveillance strategies to enhance clozapine safety in clinical practice.

## 1. Introduction

Schizophrenia is a chronic and debilitating psychiatric disorder with a multifactorial etiology involving genetic predisposition, environmental exposures, and neurodevelopmental abnormalities. Substantial evidence supports the hypothesis that these factors interact to disrupt early brain development, ultimately contributing to the pathophysiology of the disorder. Clinically, schizophrenia is characterized by a heterogeneous constellation of symptoms, including hallucinations, delusions, disorganized thought and behavior, affective flattening, apathy, and social withdrawal [1]. The onset of symptoms typically occurs in late adolescence or early adulthood and often persists throughout the patient’s lifetime, resulting in marked functional impairment and long-term disability [2]. Neurobiological models of schizophrenia implicate dysregulation in multiple neurotransmitter systems, particularly dopamine, glutamate, and serotonin. The dopaminergic hypothesis remains central, proposing hyperactivity of dopaminergic transmission in the mesolimbic pathway as a core mechanism underlying psychotic symptoms [3].

Clozapine remains the most effective antipsychotic for treatment-resistant schizophrenia (TRS), offering significant benefits in symptom control, reduction of suicidality, and overall functional improvement [4,5]. However, clozapine is not considered a first-line treatment option due to the need for close monitoring and its complex side-effect profile. Adverse effects may include sedation, weight gain, hypersalivation, seizures, myocarditis, and hematologic abnormalities such as neutropenia. While agranulocytosis was historically considered a major threat, it is now rarely fatal due to effective hematologic surveillance. Importantly, recent pharmacovigilance data indicate that pneumonia is the leading cause of death among clozapine-treated patients, followed by myocardial infarction, which is likely linked to clozapine-induced metabolic syndrome. Neuroleptic malignant syndrome, although serious, is a rare occurrence and not among the top ten causes of fatality in clozapine users [6]. Importantly, recent pharmacovigilance data indicate that pneumonia is the leading cause of death among clozapine-treated patients, followed by myocardial infarction. While metabolic syndrome associated with clozapine may contribute to cardiovascular risk, it is also possible that myocardial infarction reflects a higher baseline vulnerability in patients with TRS, independent of clozapine use [6,7].

Despite its superior efficacy, clozapine use is limited by a broad range of potentially serious adverse effects, among which myocarditis, which is a rare but potentially fatal complication. Clozapine-induced myocarditis (CIM) typically occurs within the first 2–4 weeks of treatment initiation and can present with nonspecific symptoms such as fever, fatigue, chest pain, and tachycardia [8]. Due to the overlap with clozapine’s common side effects and the often-subtle onset of myocarditis, diagnosis can be challenging. Recent literature emphasizes the importance of early recognition through systematic monitoring, including serial measurements of high-sensitivity troponin, C-reactive protein (CRP), and electrocardiographic (ECG) abnormalities. Some protocols recommend baseline and follow-up cardiac assessments during the first month of treatment to detect subclinical myocarditis and prevent progression to fulminant cardiac failure [9–11].

Although no standardized guidelines exist, growing evidence supports that timely discontinuation of clozapine upon early signs of myocarditis significantly improves patient outcomes. There is also increasing interest in identifying predictive biomarkers and developing desensitization protocols for patients who require rechallenge after CIM [12].

This study investigated the adverse impacts of clozapine on blood markers and ECG findings among patients with schizophrenia, emphasizing in early detection of life-threatening complications, such as CIM.

## 2. Materials and methods

**Fig. 1:**
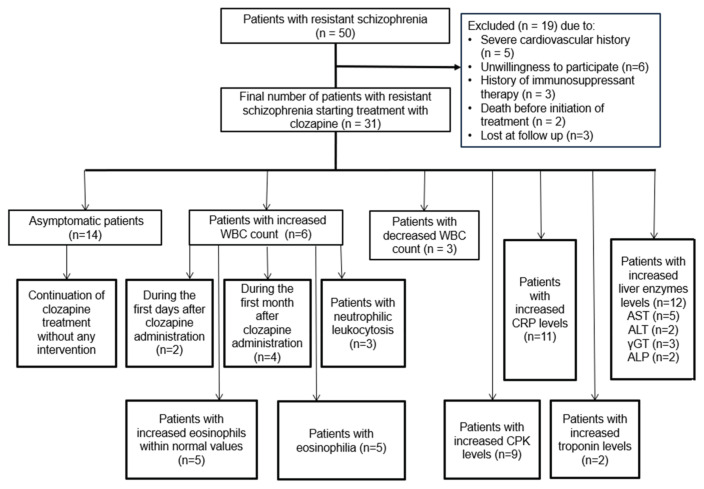
Overview of patient selection and adverse effects observed during clozapine initiation in resistant schizophrenia.

All patients treated with clozapine in a psychiatric hospital in Athens, Greece, between January 2022 and June 2024 were considered eligible for the study. We included all patients who consented to participate and had complete laboratory and clinical data available. Clozapine was initiated in 50 patients during this period; of whom 31 met inclusion criteria (see Fig. 1).

We evaluated key laboratory markers before the initiation of treatment, between days 5–10, and again after 1–2 months in patients who continued clozapine or resumed treatment at a reduced dose. Baseline and follow-up ECGs were obtained prior to clozapine initiation and on day 15.

The following parameters were recorded; white blood cell (WBC) count and differentials (neutrophils, eosinophils), C-reactive protein (CRP), hepatic enzymes (AST, ALT, ALP, GGT), creatine phosphokinase (CPK), and troponin. Normal ranges used were: WBC (4–10 K/μL), neutrophils (37–80%), eosinophils (0–7%), CRP (0–0.7 mg/dL), CPK (26–145 U/L), AST (5–33 U/L), ALT (5–34 U/L), ALP (25–125 U/L), and GGT (5–42 U/L).

Despite the publication of the international titration guideline [5], this protocol was not implemented during the study period. Consequently, the clozapine titrations used were standard rather than ancestry-based or personalized. We retrospectively reviewed titration schedules and monitored for signs of inflammatory adverse reactions. During the study period, clozapine was generally initiated at 12.5 mg once or twice daily, with daily increases of 25–50 mg, aiming for a target dose of 200–300 mg/day by week 2–3. In retrospect, this titration was faster than currently recommended by international consensus.

## 3. Results

Out of 50 patients, 19 were excluded for the following reasons; six refused consent, five had known cardiovascular disease, three were on immunosuppressants, two died before initiation, and three were discharged before follow-up labs could be collected. Thus, 31 patients were enrolled. Fourteen presented no clinically significant alterations in the evaluated markers. Six patients showed WBC elevation (two in the first days of treatment, four within the first month), and three experienced transient leukopenia that normalized without interventions. Neutrophilic leukocytosis was noted in three cases; none developed agranulocytosis. Eosinophilia was seen in five patients, primarily during the first week of titration, which may suggest early clozapine-induced inflammation [13].

Eleven patients had elevated CRP values, mostly in the first two weeks, which in retrospect should have raised concern about overly rapid titrations [14]. Elevated CPK was found in nine patients, with potential association with clozapine-induced myositis [15]. Transient elevations in hepatic enzymes were observed in five (AST), two (ALT), three (GGT), and two (ALP) patients, possibly suggesting mild clozapine-induced hepatitis [5].

Two patients (6.5%) developed myocarditis during the first three weeks of clozapine therapy, confirmed by clinical presentation (e.g., fatigue, chest pain and fever), new electrocardiographic abnormalities, and significantly elevated serum troponin levels (77.3 ng/L and 219 ng/L). No patients underwent cardiac MRI or biopsy. The diagnosis was made clinically, supported by laboratory and ECG findings. Clozapine treatment was discontinued immediately following diagnosis. Four patients developed QTc prolongation; two of them overlapped with the myocarditis cases.

## 4. Discussion

Clozapine remains the most effective antipsychotic for treatment-resistant schizophrenia, but its use is limited by rare yet severe adverse events such as agranulocytosis and myocarditis [16]. In our study, two of 31 patients (6.5%) developed CIM, a rate consistent with high-incidence settings such as Australia [17]. This incidence is alarmingly high and suggests that our titration schedule was likely too rapid.

Based on scientific literature, clozapine could possibly affect the concentration of granulocytes. It has been reported that patients under clozapine may experience elevation or decrease in total WBC and neutrophil count [18]. Although the exact mechanism of clozapine-induced neutropenia and agranulocytosis is unknown, several different suggestions have been proposed trying to explain the connection between the hematopoietic cells of bone marrow and clozapine. The most popular theory about this interaction is that clozapine is able to induce production of reactive oxygen species (ROS) leading to a powerful expression of apoptotic genes. This induces the significant destruction of granulocytes leading to increased susceptibility to bacterial and viral infections with the most common to be respiratory tract infections due to the additional involvement of clozapine into the respiratory center of the brain and medulla oblongata [19,20]. Agranulocytosis caused by clozapine is an idiosyncratic (type B) reaction, which is not dose-related so it can be potentially immune-mediated and can be associated with a toxic mechanism or both. On the other hand, at the same time, clozapine is also responsible for elevation in WBC series leading to leukocytosis. This means overproduction of WBC into the bone marrow or decreased WBC apoptosis. The most predominant theory about this phenomenon is the involvement of clozapine with pre-apoptotic genes passing again through ROS production which appears better during the first months of treatment [21,22].

Furthermore, clozapine’s metabolic side effects, including dyslipidemia, weight gain, and insulin resistance, can substantially increase cardiovascular risk. In fact, myocardial infarction ranks among the leading causes of death in clozapine-treated patients, underscoring the importance of ongoing cardiovascular monitoring and early management of metabolic syndrome [6]. Finally, direct inflammatory response triggered by clozapine itself on blood cells is an additional explanation about the changes in WBC count. As we can see, the same mechanism of action of clozapine can potentially increase or decrease WBC count based on genetic profile of each patient [23].

Several patients developed signs of systemic inflammation (elevated CRP, eosinophilia, neutrophilic leukocytosis, and increased CPK) frequently within the first two weeks. These findings support the emerging concept that rapid titration increases the risk of myocarditis, hepatitis, and myositis [24,25]. Notably, CRP elevations should have prompted caution and titration adjustments [14].

Moreover, eosinophilia is considered as another notable but less common side effect of clozapine treatment, occurring in around 1% in clozapine-treated patients [26]. This condition typically arises during the initial phase of therapy, often within the first month, and can be presented with symptoms ranging from rash to severe life-threatening drug reactions with eosinophilia and systemic symptoms (DRESS) or eosinophilic myocarditis, both of which necessitate immediate medical attention and potential discontinuation of clozapine [27]. Recent evidence also underscores that eosinophilia during clozapine titration is common but can be difficult to interpret [13]. While often benign and transient, it sometimes reflects or precedes severe hypersensitivity or inflammatory reactions, such as DRESS or myocarditis. In our cases, eosinophilia was detected in five patients early after clozapine initiation. All were asymptomatic and the eosinophilia resolved spontaneously. No patients developed DRESS, eosinophilic myocarditis, or other serious eosinophil-related organ complications. These results reinforce the need for vigilant monitoring and nuanced clinical judgment when eosinophilia is observed during clozapine therapy. Although, unlike clozapine-induced neutropenia, there are no standardized monitoring recommendations for eosinophilia, surveillance of eosinophil blood counts, including eosinophil levels, is essential to detect and manage this side effect promptly, ensuring safe and effective treatment of patients [28].

It should also be noted that increased neutrophilic leukocytosis may indicate the presence of infection, which itself is a known risk factor for the development of CIM, particularly during the titration phase. Recent reports emphasize that infection-related inflammation can potentiate myocarditis risk and may alter clozapine metabolism [25]. In our patient cohort, although some individuals exhibited transient neutrophil elevation, no active infections were clinically identified. This highlights the importance of systematic infection screening and exclusion, particularly in the presence of fever or inflammatory markers, during clozapine titration.

Furthermore, it is a fact that antipsychotic drugs may cause a transient increase in liver enzymes through two mechanisms. On the one hand, there is the idiosyncratic mechanism, where a type 1 hypersensitivity reaction with accompanying eosinophilia and an increase in autoantibodies is observed, while on the other hand, indirect liver damage is described through an increased risk of metabolic syndrome. Clozapine, among the atypical antipsychotics, is the one most associated with impaired liver function [11]. Usually, although elevations of transaminases normalize spontaneously without reducing the dose of clozapine in 50% of patients in about nine weeks, cases of severe liver damage are described in the literature. Such incidences of hepatotoxic reaction are, thankfully, rare [29,30].

Finally, a severe side effect of clozapine is CIM and cardiomyopathy. A systematic review and meta-analysis found the incidence of CIM to be approximately 0.7% and cardiomyopathy 0.6%, though rates varied widely between studies [16,31]. One explanation for this variability may be the speed of clozapine titration, since faster titration protocols have been associated with increased myocarditis risk, while slower, personalized titration schedules, potentially using C-reactive protein (CRP) monitoring, may reduce incidence and mortality [17].

We also observed several patients with elevated liver enzymes and CPK—possible markers of clozapine-induced hepatitis and myositis [5,15]. Further study is needed to determine whether these cases were part of a broader inflammatory syndrome triggered by fast titration. These observations support the implementation of cautious titration strategies and early inflammatory marker monitoring when initiating clozapine treatment. Recent international expert consensus strongly emphasizes that careful titration of clozapine is essential to minimize the risk of clozapine-induced myocarditis, underscoring the need for gradual dosage increases and routine monitoring during the initial treatment phase [24]. Implementing the international titration guideline should become a priority in the Greek psychiatric settings.

In 2025, an international panel of experts reviewed current evidence and reached widespread agreement that gradual titration of clozapine, together with systematic cardiac and inflammatory marker monitoring, is the most effective strategy to reduce the risk of CIM. Their recommendations advocate for standardized slow titration protocols internationally, reflecting updated best practice and reinforcing previous findings that rapid dose escalation is a major risk factor for myocarditis [24].

The current literature emphasizes the importance of slow, personalized titration schedules based on ancestry and metabolic status, with CRP monitoring to minimize the risk of myocarditis [5,31]. Our study did not adopt these recommendations due to institutional protocols at the time. This oversight may have contributed to the adverse outcomes observed. Therefore, our findings, including episodes of myocarditis identified timely by monitoring, further highlight the importance of these consensus-based recommendations for titration and close observation, especially during the first weeks of treatment.

Myocarditis is most likely to occur in the first 6–8 weeks of starting clozapine treatment while cardiomyopathy could arise after 9 months of treatment. Nevertheless, both conditions may transpire at any time [20]. In addition, CIM may have symptoms including hypotension, tachycardia, fever, flu-like symptoms, dyspnea and chest pain. Similar to our cases the initial cardiac events include severe elevation of troponin levels, QT prolongation and ST segment elevation similar to myocardial infarction. Such cases demand the immediate discontinuation of clozapine treatment [31]. However, there are several protocols mentioning clozapine re-challenge with slower titration rate and systematic monitoring of cardiac and inflammatory markers [32]. Our findings, that 6.5% of patients developed myocarditis, mirror high-incidence settings and underscore the importance of implementing slower titration protocols. This rate is considered high by international standards [5,17,24,31,32]. In retrospect, we recognize that our hospital’s titration protocol, employing rapid dose increases, likely contributed significantly to this outcome, consistent with the emerging literature linking rapid titration, inadequate inflammatory marker surveillance, and increased CIM risk [13,25]. These results support recent global guidelines calling for more gradual dose increments and robust inflammatory marker surveillance, both to reduce myocarditis risk and to permit earlier intervention if warning signs appear [13,24,25]. Comparative evaluation of our titration practices against international guidelines is essential to improving safety in clozapine prescribing in Greece.

Since completing the study, we have updated local protocols in accordance with international guidelines, implementing slower, personalized titration schedules as well as systematic baseline and weekly monitoring of CRP and troponin during the first month of treatment. Staff training has also been enhanced to ensure timely detection and appropriate intervention to early inflammatory signs such as fever, CRP elevation, or eosinophilia. Our experience highlights the necessity of continuous medical education, critical reflection, and swift adoption of evidence-based practice changes as new data and international consensus emerge. We offer this article not only as new scientific data but, more importantly, as a warning and learning opportunity.

Recent regulatory changes have further complicated the safety landscape for clozapine. In June 2025, the United States Food and Drug Administration officially discontinued the Risk Evaluation and Mitigation Strategy (REMS) for clozapine, citing significant barriers to access and administrative burdens without clear improvement in patient outcomes [33]. While this decision may improve access, it also places greater responsibility on clinicians to implement robust, individualized monitoring protocols to detect hematologic and cardiac complications early, especially given the rare but serious adverse events observed in our study.

## 5. Conclusion

In conclusion, clozapine is an important and powerful second generation anti-psychotic drug used in a variety of patients, mostly in those with treatment resistant schizophrenia with excellent therapeutic results. Nevertheless, there are multiple side effects which may cause significant complications and require termination of clozapine therapy. This study included patients from psychiatric hospital of Attica treated with clozapine and we found that 2 patients out of 31 had CIM while the rest of them had no significant complications. Systematic hematological and cardiological monitoring is necessary to evaluate the progress of therapy and early diagnose possible adverse effects.

## Data Availability

Data associated are available upon reasonable request from the authors.
